# Adipose tissue distribution and metabolic profile of young individuals with turner syndrome

**DOI:** 10.1210/jendso/bvag016

**Published:** 2026-01-27

**Authors:** Varsha Mary Thomas, Philippe Backeljauw, Amy Sanghavi Shah, Katherine Bowers, Iris Gutmark-Little

**Affiliations:** Division of Endocrinology, Cincinnati Children's Hospital Medical Center and the University of Cincinnati, Cincinnati, OH 45229, USA; Division of Endocrinology, Cincinnati Children's Hospital Medical Center and the University of Cincinnati, Cincinnati, OH 45229, USA; Division of Endocrinology, Cincinnati Children's Hospital Medical Center and the University of Cincinnati, Cincinnati, OH 45229, USA; Division of Endocrinology, Cincinnati Children's Hospital Medical Center and the University of Cincinnati, Cincinnati, OH 45229, USA; Division of Endocrinology, Cincinnati Children's Hospital Medical Center and the University of Cincinnati, Cincinnati, OH 45229, USA

**Keywords:** Turner syndrome, body composition, bioelectrical impedance, visceral adiposity, DXA, obesity

## Abstract

**Objective:**

Turner syndrome (TS) is the most common sex chromosome abnormality in females. Incidence of cardiometabolic complications is high in TS. Obesity and high visceral adiposity (VA) are additional risk factors. Body mass index (BMI) is not the best index of adiposity in TS due to short stature. Dual-energy x-ray absorptiometry (DXA) scan and bioelectrical impedance (BI) could be alternatives. The primary objective is to evaluate alternative methods for body composition assessment in youth with TS. The secondary goal is to characterize body composition and metabolic profiles of youth with TS compared with youth without TS.

**Methods:**

This is a cross-sectional study. Participants with TS are on ≥50 mcg transdermal estradiol equivalent replacement or spontaneously menstruating. The control group consisted of youth with normal/lean BMI and obese BMI.

**Results:**

Twenty-seven TS, 15 obese, and 15 lean youth were recruited (13-19 years; female). In the TS group, correlation is noted between DXA and BI for fat mass (FM), lean mass (LM), and VA. VA (356.56 +/−225.82 g) and FM (27.06 +/−11.39 g) from DXA were higher for the TS group compared to the lean group's VA (162.66 +/−68.58 g) and FM (19.44 +/−19.47 g), even with comparable LM and total weight. The prevalence of dyslipidemia was 38% in the TS group. The lean group had no dyslipidemia.

**Conclusion:**

Differences in LM, FM, and VA would not have been identified by BMI alone. BI may be a convenient and noninvasive outpatient tool in TS to assess body composition and identify risk factors for metabolic dysfunction, such as low LM and high FM.

Turner syndrome (TS) is the most common sex chromosome abnormality in females and occurs in approximately 1 in 2000 live female births [1]. Individuals with TS have increased morbidity and mortality compared with the general population [2-4]. Cardiovascular disease is a leading cause of mortality [2, 5, 6]. There is also an increased prevalence of obesity [7, 8] and comorbidities, including type 2 diabetes mellitus [9, 10], hypertension [11, 12], and dyslipidemia [2, 3, 13-17].

Individuals with TS are known to have a higher body mass index (BMI). They also are known to have increased visceral adiposity and waist circumference, along with lower lean mass and skeletal mass compared to age-matched peers [18, 19]. The current best practice for patients with TS is to assess BMI as an indicator of adiposity at every clinical visit. However, BMI is not a good screening tool for obesity in individuals with short stature because, given the prevalence of short stature seen in TS, the use of BMI in the TS population can be particularly misleading [20]. Furthermore, BMI reflects only total body weight and does not delineate lean mass and fat mass (FM) [21].

It is well-documented that increased waist circumference and abdominal obesity are associated with cardiometabolic risk [22]. Methods such as dual-energy x-ray absorptiometry (DXA) scan and bioelectrical impedance (BI) provide more precise information about body composition and the distribution of adiposity [23]. A recent study by Mondal et al compared body composition in adolescents and young adults with TS with healthy age-matched controls using DXA scan and reported a higher total body fat percentage and lower lean mass in the TS group. They also reported that altered body composition increased risk for cardiometabolic complications [18]. Similarly, Gravholt et al compared body composition data from DXA scans in 54 adult TS women with age-matched controls and reported that patients with TS have lower lean body mass and increased total and visceral FM [19].

DXA scans are the gold standard for measuring body composition, but they cannot be used for routine monitoring due to their prohibitive cost, concern for repeated radiation exposure, and limited availability. It is important to identify alternate methods for measuring body composition. BI has the advantage of being less expensive, incurring no radiation exposure, and having easier accessibility in a clinical setting. However, before BI can be used in the clinical setting to measure body composition, it is important to compare data from BI to an accepted standard such as DXA scan. To the best of our knowledge, no studies have been done in the pediatric TS population comparing DXA and BI data where unique differences in body composition are known to exist. We aimed to assess body composition in youth with TS using DXA and BI and then compare adiposity indices between the 2 techniques. If a strong correlation between DXA and BI exists, BI could be a valuable tool in the clinical setting to assess adiposity. As a secondary goal, we planned to characterize the metabolic profile of youth with TS and compare it to normal-weight and obese youth without TS.

## Materials and methods

### Study participants

Three groups were recruited: youth with TS and 2 comparison groups (normal/lean BMI and youth with obese BMI). Individuals with TS were identified from patients at Cincinnati Children's Hospital Medical Center (CCHMC). The control groups were recruited as part of an ongoing study in adolescents, Understanding the Impact of Youth Onset Obesity and Type 2 Diabetes on the Neurovascular Unit (R01NS125316). All participants are biological females.

In all 3 groups, an age range of 13 to 19 years was used as an inclusion criterion. This was chosen in the context that puberty is a time of increased insulin resistance and major changes in body composition [24]. Earlier identification of at-risk individuals before adulthood will aid in primary prevention as well. All participants in the TS study group were treated with at least 50 mcg transdermal β-estradiol or its equivalent for hormone replacement therapy (HRT) or were spontaneously menstruating. Obese control group participants all had BMI ≥95th percentile, and the normal/lean control group had a BMI between the 5th and 85th percentiles. An existing diagnosis of diabetes mellitus was used as an exclusion criterion for all 3 groups.

Written informed consent/assent/parental permission was obtained from participants/parents/legal guardians. The use of clinical information for research purposes and the protection of privacy were in compliance with the Health Insurance Portability and Accountability Act requirements.

### Methods

All participants had a single in-person study visit, which involved anthropometric measures, phlebotomy, and imaging. Height and weight were measured in all 3 groups. Lipid profile, liver enzymes, inflammatory markers, hemoglobin A1c (HgbA1c), DXA, and BI were obtained in all 3 groups. Measurement of iliac waist and hip circumference and sagittal abdominal diameter was done only in the TS group. Weight, sagittal abdominal diameter, and waist and hip circumference were measured to the 100th decimal place. Height was measured to the 10th decimal place. Waist circumference was measured per National Health and Nutrition Examination Survey (NHANES) National Youth Fitness Survey Body Measures Procedures Manual. Sagittal abdominal diameter was recorded to be the distance between the back and the highest point of the abdomen using calipers. The same 2 weight scales and stadiometers were used for the TS study group, and all anthropometric measurements were done by three team members to limit variability in measuring techniques.

Electronic medical records were reviewed to obtain demographics (age and race). In the TS group, karyotypes and details of the current medication regimen, including HRT, were also reviewed. In the TS group, if TS study participants had annual screening blood work done for clinical purposes (liver enzymes, lipid profile, and HgbA1c) within 12 weeks before the research visit, then they were not repeated. C-reactive protein (CRP) and interleukin-6 (IL-6) were obtained for research purposes. Metabolic lab values were collected only from 26 TS group participants because 1 participant declined lab draw on the day of the visit. There was 1 participant in the TS group who was not fasting. The control group participants were all fasting. Two participants in the lean group and 1 participant in the obese group did not have all the metabolic lab parameters due to insufficient sample/refusal of blood draw. All lab assays were run at the CCHMC main lab for the TS study group, except for 2 participants who had done the blood draw at an outside facility. All participants completed their study visit.

DXA scans were done using Hologic Horizon® [25]. The BI scan was done using the InBody 570® body composition analyzer [26]. Both imaging modalities were completed by trained medical staff per manufacturers’ guidelines. FM, lean mass, body weight, body fat percentage, and visceral fat were selected as body composition indices compared between the 3 groups. As detailed in Table 1, lean mass by BI was calculated by adding segmental data for the upper limbs, trunk, and lower limbs together; this was compared with lean mass without head by DXA. DXA FM was compared to body FM by BI. Similarly, the total mass by DXA was compared to weight by BI. The total body fat percentage by DXA was compared with the percentage body fat obtained by BI, and finally, visceral adiposity measures between both techniques were compared. Except for visceral adiposity measures, all other indices had similar units. The estimated visceral adipose tissue by DXA (in grams) was compared to the visceral fat level obtained by BI. The visceral fat reported by BI is a whole number without units. Per the manufacturer’s guidelines, a visceral fat level of 10 on BI equates to 100 cm² of visceral fat. In the obese group, 13 individuals completed BI, and 15 individuals completed DXA scans. All participants in the TS and lean control groups completed both imaging modalities. Lean mass index (lean mass/height^2^) and FM index (FM mass/height^2^) were calculated to adjust for height difference between the 3 groups (Table 1) [27].

**Table 1:** Comparison of body composition indices between the 2 techniques

	DXA	BI
Fat mass index	Fat mass (kg/m²)	Body fat mass (kg/m²)
Lean mass index	Lean mass (kg/m²)	Segmental lean analysis (upper limbs+ trunk+ lower limbs) (kg/m²)
Total mass	Total mass including head (g)	Body weight (g)
Percentage of adiposity	Total percent body fat (%)	Percent body fat (%)
Visceral fat marker	Estimated visceral adipose tissue (g)	Visceral fat level (no units)

### Statistical analysis

All data were analyzed using SAS® software and Microsoft Excel®. Demographic and anthropometric measures were compared between TS, obese, and lean controls. Continuous variables were summarized with means and SD, and categorical variables were summarized with counts and percentages. Differences between groups (TS vs lean and TS vs obese) were assessed using a 2-sided Wilcoxon rank test for continuous variables. *P*-values were not generated for race since it is clearly White predominant. Fat and lean mass indices obtained from DXA and BI scans were correlated across study groups using Spearman correlation coefficients, including *P*-values and 95% confidence intervals. Correlations between DXA and BI were visualized on scatter plots for measures of FM, lean mass, body fat percentage, and visceral fat. In addition, the agreement between DXA and BI for measures of FM, lean mass, and total weight was assessed using Bland-Altman plots. The mean difference and the SD of these differences were computed to determine the 95% limits of agreement. These limits were calculated as the mean difference ±2 SD of the differences. Additional anthropometric measures were correlated with visceral fat measures obtained from DXA and BI within the TS sample. Measures were summarized with means and SD and correlated using Spearman correlation coefficients.

Continuous measures of metabolic markers were compared across groups using a Kruskal-Wallis test. The presence of dyslipidemia and elevated CRP and IL-6 were compared across groups using a Fisher's exact test. CRP and IL-6 were converted to categorical covariates with normal levels designated as 0 and abnormal as 1. CRP and IL-6 normal values were determined based on the reference range for the CCHMC laboratory. For IL-6, normal is defined as ≤3, and for CRP, it is ≤0.4. CCHMC laboratory reports normal values as within reference range and not as a whole number, which makes it difficult to analyze them continuously. Hence, they were analyzed categorically. In the TS group, 1 participant had an HgbA1c reported less than 4.6%, so a value of 4.5% was used for statistical analyses. Similarly, 2 participants had alanine aminotransferase reported as less than 7; therefore, a value of 6 was used in statistical analyses. Dyslipidemia was defined as total cholesterol (TC) ≥200 mg/dL, low-density lipoprotein (LDL) ≥130 mg/dL, triglycerides (TG) ≥130 mg/dL, or high-density lipoprotein (HDL) <40 mg/dL [28].

## Results

### Demographics, anthropometry, and other clinical information

Twenty-seven female participants were recruited for the TS group. The control group had 30 female participants, 15 classified as lean and 15 classified as obese based on BMI. The 3 groups were comparable in age (Table 2). Race and ethnicity were predominantly (>90%) White and non-Hispanic in all three groups. There is a statistically significant difference in height, weight, and BMI between TS and the obese group. When compared to the lean group, the TS group had a statistically significant difference in height and BMI but not in total weight, as detailed in Table 2. Obese youth had significantly higher BMI and BMI Z-scores compared to the individuals with TS and lean youth as expected (Table 2). Within the TS study group, 10 participants (37%) were noted to have lymphedema of varying degrees based on clinical exam.

**Table 2:** Demographic and anthropometric data

	TS study group (n = 27)	Obese youth (n = 15)	*P-*value	Lean youth (n = 15)	*P-*value
Demographic					
White race (%)	n = 25 (93)	n = 12 (80)		n = 13 (87)	
Other race (%)	n = 2 (7)	n = 3 (20)		n = 2 (13)	
Age, years, mean (SD)/median (IQR)	16.41 (1.8)/16.00 (15.00-18.00)	16.27 (1.3)/16.00 (15.00-17.00)	.85	16.40 (2.41)/16.00 (14.00-19.00)	1.0
Anthropometric, mean (SD)/median (IQR)					
Height, cm	150.82 (6.26)/1.51.0 (146.60-154.20)	163.73 (76.98)/162.20 (160.80-166.00)	<.0001	160.15 (7.60)/160.40 (155.20-166.50)	.002
Height Z-score	−1.75 (0.93)/−1.68 (−2.14-−1.18)	0.19 (1.10)/0.01 (−0.33-0.59)	<.0001	−0.24 (1.17)/−0.15 (−0.73-0.58)	.008
Weight, kg	62.04 (17.30)/60.2 (47.50-67.30)	103.78 (18.02)/104.40 (96.60-110.50)	<.0001	55.49 (8.71)/57.20 (58.90-60.70)	.25
BMI, kg/m^2^	27.27 (7.40)/26.8 (19.98-30.05)	38.61 (5.37)/36.24 (35.06-44.52)	.0001	21.65 (3.18)/21.26 (18.61-25.13)	.01
BMI Z-score	1.05 (1.23)/1.40 (0.02-1.72)	2.52 (0.62)/2.35 (1.90-3.16)	.0002	0.20 (0.82)/−0.06 (−0.48-0.96)	.008
DXA, mean (SD)/median (IQR)					
Fat mass index, kg/m²	12.00 (5.06)/11.8 (7.40-15.01)	18.82 (4.29)/17.91 (14.95-23.73)	.0005	7.58 (1.93)/7.36 (6.13-0.90)	.007
Lean mass index, kg/m²	13.73 (2.39)/13.87 (11.85-14.69)	17.62 (1.76)/18.08 (16.17-18.90)	.0001	12.24 (1.83)/12.04 (10.80-13.75)	.05
Total mass, kg	61.8 (17.34)/59.72 (46.74-66.05)	103.36 (18.03)/102.75 (95.83-112.01)	<.0001	55.26 (8.74)/56.64 (48.25-59.96)	.45
Body fat percentage, %	40.74 (7.12)/42.50 (36.40-45.30)	48.69 (5.01)/49.40 (45.10-53.00)	.001	35.20 (5.16)/35.10 (30.20-38.20)	.02
Visceral adipose tissue, g	356.56 (225.82)/297.00 (154.00-477.00)	591.13 (221.69)/550.00 (403.00-718.00)	.004	162.66 (68.58)/154.00 (120.00-202.00)	.004

Two-sided Wilcoxon rank test.

Abbreviations: BI, bioelectrical impedance; BMI, body mass index; DXA, dual-energy x-ray absorptiometry; IQR, interquartile range; TS, Turner syndrome.

As detailed in Table 3, 16 participants in the TS group have primary ovarian insufficiency and were on HRT. The remaining 11 have spontaneous menarche and regular menstrual cycles. Eight participants have 45,X, and 6 participants have mosaic TS karyotypes. The remaining 13 individuals have karyotypes other than 45,X and mosaic 45, X/46, XX (Table 3). Four participants had never been on GH. One participant was on metformin for obesity. Other nonendocrine medications included aspirin (n = 1), atenolol (n = 2), losartan (n = 1), amlodipine (n = 1), fluoxetine (n = 1), viloxazine (n = 1), dexmethylphenidate (n = 1), and lisdexamfetamine (n = 3). None of the control group participants were on any medications.

**Table 3:** Karyotype and medication information for TS study participants

TS study group (n = 27)	Karyotype	HRT	Endocrine pertinent medications
	45,X: n = 8 45,X/46, XX: n = 6 Other karyotypes: n = 13*^a^*	Transdermal estradiol: n = 9 Oral estrogen: n = 7 No HRT: n = 11	On levothyroxine: n = 7 Prior GH therapy: n = 22 On GH: n = 1 Metformin: n = 1

Other karyotypes included 45,X/46,XX,del Xp21; 46,X,r(X)(p11.21); 45,X/46,Xr(X); 45,X/46,isoX(q10); 46,X,r(X)(p22.3q28)/45,X; 45,X/46,X,idlc(Y); 46,X del(Xp22.1); 45,X,t(8;15)(q24.22;q21.2)/46,X,r(X) (p22.33q22.1), t(8;15)/47,X,t(8;15), +2mar/46,X,t(8;15), +mar45,X/46,X,i(X)(q10), 45,X/46,XY, and 3 participants with 45,X/47,XXX.

Abbreviations: HRT, hormone replacement therapy; TS, Turner syndrome.

### Body composition studies

Lean mass and fast mass were adjusted for height (Table 2). The TS and lean groups have a comparable lean mass index; however, the TS group has a higher FM index compared with the lean group. Even with comparable lean mass and total weight, the visceral adiposity is much higher for the TS group compared to lean group. The TS group has a mean total mass (∼60 kg) like the lean control group's total mass (∼56 kg), but the mean visceral adipose tissue mass in the TS group (∼356 g) was more than double the amount in the lean group (∼162 g). As expected, the obese group had a higher value for all body composition indices using DXA when compared to the TS group (Table 2).

**Figure 1:**
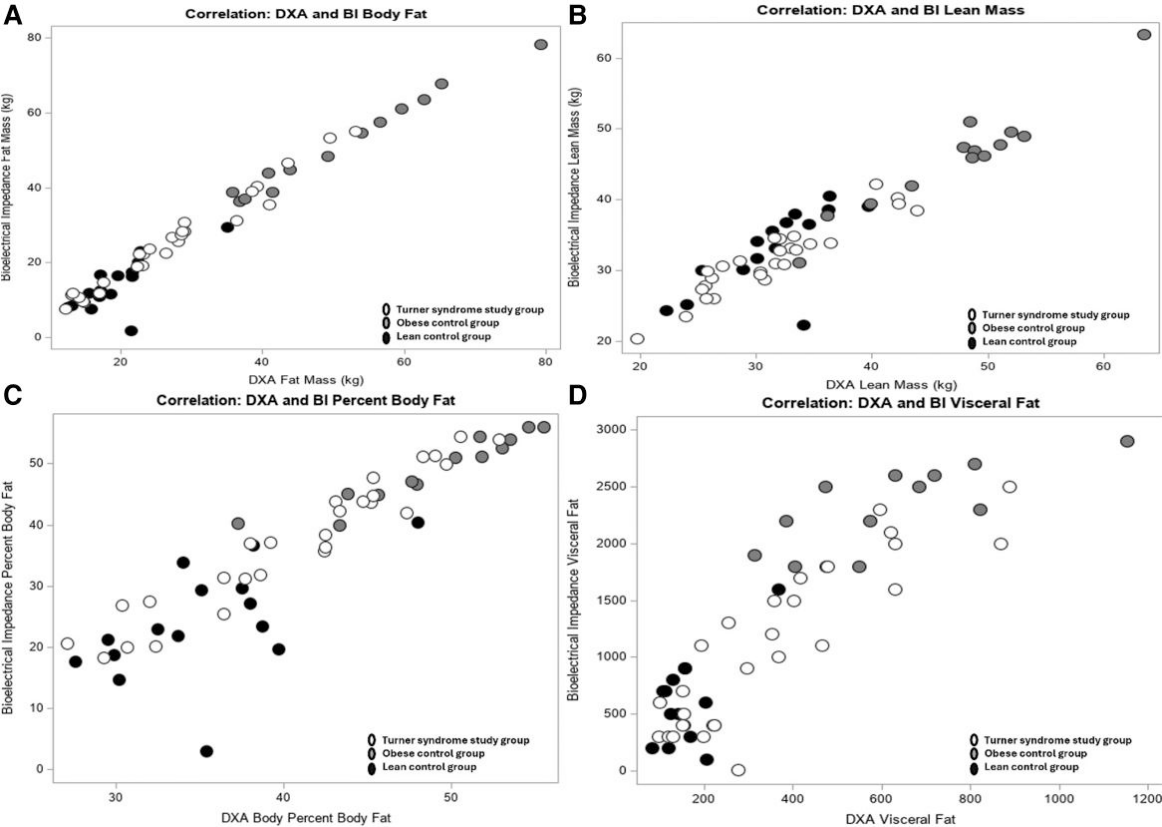
Turner syndrome study group (white data points). Obese control group (grey data points). Lean control group (black data points). Correlation between dual-energy x-ray absorptiometry scan and bioelectrical impedance data for body fat mass (A), lean mass (B), percentage body fat (C), and visceral fat markers (D) for the Turner syndrome study group (white data points), obese control group (grey data points), and lean control group (black data points).

**Figure 2:**
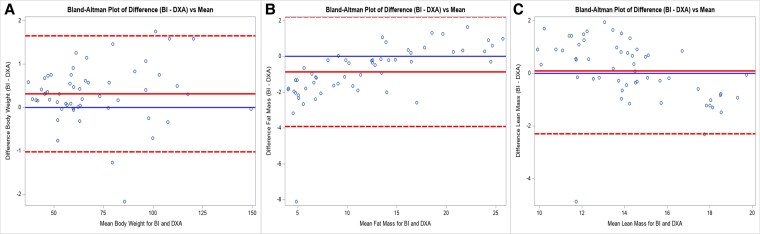
Bland-Altman plots comparing mean total mass (A), fat mass (B) and lean mass (C) between dual-energy x-ray absorptiometry scan and bioelectrical impedance for the Turner syndrome study group, obese control group, and lean control group.

As noted in Table 4, Spearman's correlation analysis showed a positive correlation between BI and DXA scan parameters in all 3 groups. In the TS study group, a statistically significant correlation was noted between DXA and BI for all body composition parameters. In the obese group, a positive correlation was noted as well, but it was less strong for visceral fat markers. In the lean group, a comparatively weaker correlation was noted between DXA and BI. Additionally, the visceral fat marker correlation was not statistically significant between the 2 techniques (Table 4). Scatter plot data showing the correlation between various measures of body composition using DXA and BI are shown in Fig. 1 for all 3 groups. A Bland-Altman plot of agreement for total weight, FM, and lean mass is shown in Fig. 2. Visceral adiposity is not compared using a Bland-Altman plot since units are different. Two methods have good agreement with regard to weight. For lean mass, there is some variation, but they are mostly in agreement. For FM, the data points seem to be less in agreement for individuals with increased adiposity.

**Table 4:** Spearman correlation of body composition indices using DXA and BI

	TS study group	Obese youth	Lean youth
	DXA n = 27	DXA n = 15	DXA n = 15
	BI n = 27	BI n = 13	BI n = 15
DXA fat mass index vs BI fat mass index	r = 0.99 *P*-value <.0001 95% CI: 0.97, 0.99	r = 0.0.98 *P*-value <.0001 95% CI: 0.94, 0.99	r = 0.70 *P*-value .003 95% CI: 0.28, 0.89
DXA lean mass index vs BI lean mass index	r = 0.88 *P*-value <.0001 95% CI: 0.74, 0.94	r = 0.96 *P*-value <.0001 95% CI: 0.84, 0.99	r = 0.60 *P*-value .02 95% CI: 0.11, 0.84
DXA body weight vs BI body weight	r = 0.99 *P*-value <.0001 95% CI: 0.99, 0.998	r = 0.99 *P*-value <.0001 95% CI: 0.96, 0.997	r = 0.99 *P*-value <.0001 95% CI: 0.97, 0.997
DXA body fat percent vs BI body fat percent	r = 0.96 *P*-value <.0001 95% CI: 0.91, 0.98	r = 0.95 *P*-value <.0001 95% CI: 0.82, 0.98	r = 0.55 *P*-value .03 95% CI: 0.04, 0.82
DXA visceral fat mass vs BI fat level	r = 0.85 *P*-value <.001 95% CI: 0.69, 0.93	r = 0.72 *P*-value .006 95% CI: 0.24, 0.90	r = 0.12 *P*-value .66 95% CI: −0.42, 0.60

Abbreviations: BI, bioelectrical impedance; BMI, body mass index; CI, confidence interval; DXA, dual-energy x-ray absorptiometry; TS, Turner syndrome.

In the TS group, iliac waist circumference, hip circumference, and sagittal abdominal diameter showed a positive correlation when compared with visceral fat measures by both DXA and BI (Table 5).

**Table 5:** Mean and Spearman correlations utilizing other adiposity measures relative to DXA and BI visceral adipose tissue in the TS study group

TS study group n = 27	Mean (SD)	Correlation with DXA visceral adipose tissue	Correlation with BI visceral fat level
Iliac waist circumference, cm	87.04 (16.97)	r = 0.89 *P*-value <.0001	r = 0.92 *P*-value <.0001
Hip circumference, cm	96.67 (12.48)	r = 0.76 *P*-value <.0001	r = 0.89 *P*-value <.0001
Sagittal abdominal diameter, cm	19.23 (3.91)	r = 0.89 *P*-value <.0001	r = 0.84 *P*-value <.0001

Abbreviations: BI, bioelectrical impedance; BMI, body mass index; DXA, dual-energy x-ray absorptiometry; TS, Turner syndrome.

### Metabolic laboratory values

The prevalence of dyslipidemia was 38% in the TS group, with a lipid panel showing elevated TC (15%), elevated LDL (8%), elevated TG (27%), and low HDL (15%). Among the obese group participants, the prevalence of dyslipidemia was 62%, with elevated TG (23%) and low HDL (54%). TC or LDL were not elevated in the obese group. The lean control group did not have any dyslipidemia (Table 6). There was a statistically significant difference between TG and HDL between the 3 groups. There was 1 participant in the TS group who was not fasting and had a normal TG of 52 mg/dL. Liver enzymes were increased in the TS group. All 3 groups had normal mean HgbA1c. CRP and IL-6 were not different between the groups (Table 6).

**Table 6:** Demographic, anthropometric, metabolic, and inflammatory markers across study groups

	TS study group	Obese youth	Lean youth	*P*-value
	(n = 26)	(n = 15)	(n = 15)	
Metabolic markers^1^				
Dyslipidemia present (%)	n = 10 (38)	n = 8 (53)	n = 0	.0002
LDL, mg/dL, mean (SD)	85.56 (33.52)	96.46 (17.29)	83.26 (20.01)	.371
HDL, mg/dL, mean (SD)	52.46 (11.99)	40.80 (10.30)	55.73 (11.27)	<.001
TG, mg/dL, mean (SD)	97.00 (47.83)	108.00 (43.23)	61.33 (20.61)	<.001
TC, mg/dL, mean (SD)	161.50 (35.09)	158.23 (22.65)	142.66 (31.15)	.26
HgbA1c, %, mean (SD)	5.2 (0.25)	5.30*^a^*(0.17)	5.17 (0.175)	.12
AST, units/L, mean (SD)	28.15 (13.90)	18.80 (3.67)	19.23*^b^* (8.04)	.003
ALT, units/L, mean (SD)	25.77 (17.49)	17.80 (6.39)	13.00^2^ (11.08)	.004
Inflammatory markers^2^				
Elevated CRP	Abnormal n = 7	Abnormal n = 4	Abnormal: n = 1	.37
	Normal n = 20	Normal n = 11	Normal: n = 13	
Elevated IL-6	Abnormal n = 1	Abnormal n = 1	Abnormal: n = 3	.21
	Normal n = 26	Normal n = 11	Normal n = 11	

Continuous variables compared using the Kruskal-Wallis test^1^ and categorical variables using Fisher's exact test^2^.

Abbreviations: ALT, alanine aminotransferase; AST, aspartate aminotransferase; CRP, C-reactive protein; HDL, high-density lipoprotein; HgbA1c, hemoglobin A1c; IL-6, interleukin-6; LDL, low-density lipoprotein; TC, total cholesterol; TG, triglycerides; TS, Turner syndrome.

^*a*^n = 14.

^*b*^n = 13.

## Discussion

Our study aimed to assess body composition utilizing both DXA and BI in adolescents and youth with TS. Our analysis shows that DXA and BI data correlate well in the TS study group. Both imaging modalities provided information about body composition, which would not have been obtained by BMI alone. TS participants had higher total fat and visceral mass than the lean group but not more than the obese group. Both studies had a good correlation between the TS study groups for body composition. A Bland-Altman plot shows that total weight and lean mass measurements have good agreement between BI and DXA methods, with only a slight deviation at higher values. FM measurements have more variability and less agreement, particularly at higher FM values. The prevalence of elevated liver enzymes was higher in the TS group compared with the control groups. Dyslipidemia was higher in the TS group compared with the lean group.

Multiple previous studies have reported abnormal body composition in both the pediatric and adult TS population, including lower lean mass and increased visceral adiposity. Accurately assessing body composition could be helpful in individuals with TS, considering the increased risk for cardiovascular morbidity and mortality. Ostberg et al compared magnetic resonance imaging and BI data in 6 adult estrogen-treated women with TS to age-matched healthy controls and reported an excess of total and visceral adipose tissue [29]. Mondal et al assessed body composition in adolescents and young adults with TS using DXA [18]. Similarly, Gravholt et al studied body composition using DXA scans in 54 adult TS women [19]. However, these studies used DXA or other imaging modalities that might not be easily available. There is limited data available regarding BI data in the pediatric TS population and no data correlating BI and DXA scan data in the pediatric TS population.

A DXA scan is a good tool to assess body composition, but it is not an optimal tool for frequent monitoring in the outpatient setting. This is because of limited availability in the outpatient setting, the need for staff with advanced training, and the risk for radiation exposure, though this exposure is typically low. On the other hand, BI is convenient, the device takes about the same space as a regular scale, and it can easily be used by outpatient clinic staff during regular clinic visits without advanced training and has no radiation exposure. We found a strong and statistically significant correlation between the 2 techniques in individuals with TS. However, based on Bland-Altman data, BI tends to overestimate FM and underestimate lean mass in individuals for higher obesity. BI may not be an optimal tool for precise individual assessment. Nonetheless, BI may serve as a practical tool for monitoring adiposity trends over a period in individuals with TS.

The TS group has almost double the amount of visceral FM compared with the lean group even though both groups had similar mean total mass and lean mass. Based on 2009 DXA Composition Reference Values from NHANES, the median fat percentage for 16-year-old White females is 32.5% (SD 6.68%) [30]. The median total body fat percentage in our TS study group was 42.5%. Higher body fat percentage, particularly higher visceral fat, is known to be a risk factor for poor cardiovascular outcomes. Current clinical practice uses weight and BMI as indicators of obesity; it is notable that our TS group had a mean BMI of 27 kg/m² with a normal average BMI Z-score but had a higher FM index than lean individuals. Therefore, total mass and BMI may not be sufficient to understand body composition and visceral adiposity and identify at-risk individuals. This is particularly relevant in the pediatric age group, where primary prevention is possible.

Our analysis showed that iliac waist circumference, hip circumference, and sagittal abdominal diameter correlated with visceral FM by DXA and visceral fat level from BI. According to NHANES data [31], the mean waist circumference in 16-year-old girls in the United States from 2015 to 2018 was 82.2 cm; the mean waist circumference in our TS group was slightly higher at 87 cm. Additionally, the mean sagittal abdominal diameter in 16-year-old females from NHANES data from 2011 to 2014 was 18.8 cm [32]; the mean in our TS group is slightly higher at 19.23 cm. Our data show that these truncal obesity measures correlate with visceral adiposity. Adding these measures to clinical practice could provide additional information about body composition. However, waist circumference, sagittal abdominal diameter, and hip circumference require specific expertise to allow accurate identification of landmarks for precise measurement. This introduces potential errors. The measurements were also time-consuming because each participant had to agree to be measured in a private area with a chaperone present to ensure these landmarks were properly marked. At least 2 medical staff are needed for proper measurement. The time, training, and precision required for this may make these methods less feasible for routine use in all clinical settings. BI is a user-friendly tool, making it potentially easier to implement in clinical settings.

Individuals in the TS group also had a higher prevalence of dyslipidemia compared to the lean group, despite a comparable mean total mass. TS participants were noted to have elevations in all 4 cholesterol markers, but in the obese group, abnormal values were notable only for TG and HDL. Even with significantly higher BMI in the obese group, individuals with TS have a higher degree of metabolic risk and warrant closer follow-up. Additionally, liver enzymes were higher in the TS study group. This has been well-described in the TS population and has been postulated to be related to obesity, insulin resistance, autoimmune pathology, or steatohepatitis.

Previous studies reported increased inflammatory markers in individuals with TS [33, 34]. In the general population, obesity is known to be associated with increased inflammatory markers such as CRP and IL-6 [35]. Elevation of CRP and IL-6 may predict the development of type 2 diabetes mellitus [36]. Studies in the adult TS population have shown similar findings and have reported that markers of adiposity, such as IL-6, adiponectin, CRP, TNF-α, and leptin, are elevated [33, 34]. However, we did not find statistically significant differences between the 3 groups regarding the inflammatory markers measured. Ostberg et al [34] reported increased IL-6 and CRP in adult obese women with TS. Prospective studies will be needed to assess whether a state of low-grade inflammation is seen with increasing age in TS and how this is connected to cardiometabolic morbidity.

Limitations of our study include a smaller sample size and a predominantly non-Hispanic White study population. Therefore, we are unable to address racial and ethnic differences. In the TS group, variable degrees of lymphedema were noted in approximately one-third of the study group. We are unable to determine whether the presence of lymphedema significantly affected the measurements of body composition. Additional studies with a larger cohort would be needed to address these limitations. Due to the inherent differences in body structure or proportionality, it is challenging to identify a well-matched control group. Additionally, the control group data in this study were obtained from a separate study, which presents a limitation in terms of direct comparability.

In conclusion, our data indicate that BI is a convenient, noninvasive, and accurate tool to assess body composition in TS. Considering the increased cardiometabolic risk, morbidity, and mortality in TS, such clinical evaluation of body composition could provide valuable additional information to identify those at risk for metabolic dysfunction.

## Data Availability

Restrictions apply to the availability of some or all data generated or analyzed during this study to preserve patient confidentiality or because they were used under license. The corresponding author will, on request, detail the restrictions and any conditions under which access to some data may be provided.
